# Supplementary material for: Inhibition of RNA Binding in SND1 Increases the Levels of miR-1-3p and Sensitizes Cancer Cells to Navitoclax
Source: Cancers (Basel). 2022 Jun 24;14(13):3100. doi: 10.3390/cancers14133100 (PMC9265050; doi:10.3390/cancers14133100)
Supplement: Supplementary file 1 [file cancers-14-03100-s001.zip › Supplenmentary figures.pdf]

Supplementary Materials: Inhibition of RNA Binding in SND1 Increases the Levels of miR-1-3p and Sensitizes Cancer Cells to Navitoclax

Saara Lehmusvaara, Teemu Haikarainen, Juha Saarikettu, Guillermo Martinez Nieto and Olli Silvennoinen

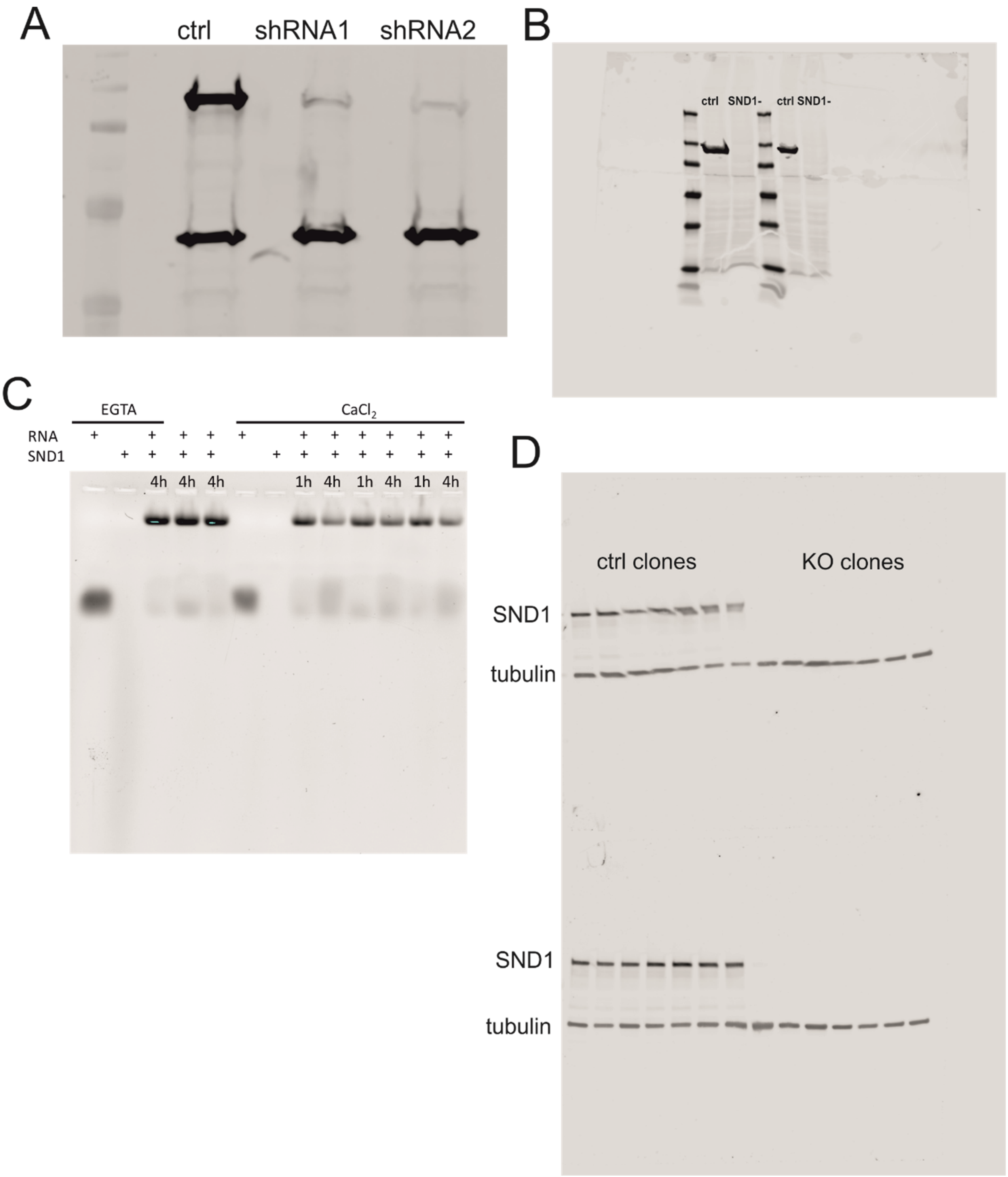

Figure S1. Original Western blot (A, B), PAGE gel (C) and Western blot (D) pictures, related to Figures 1A, 2F, 3A and 5E respectfully.

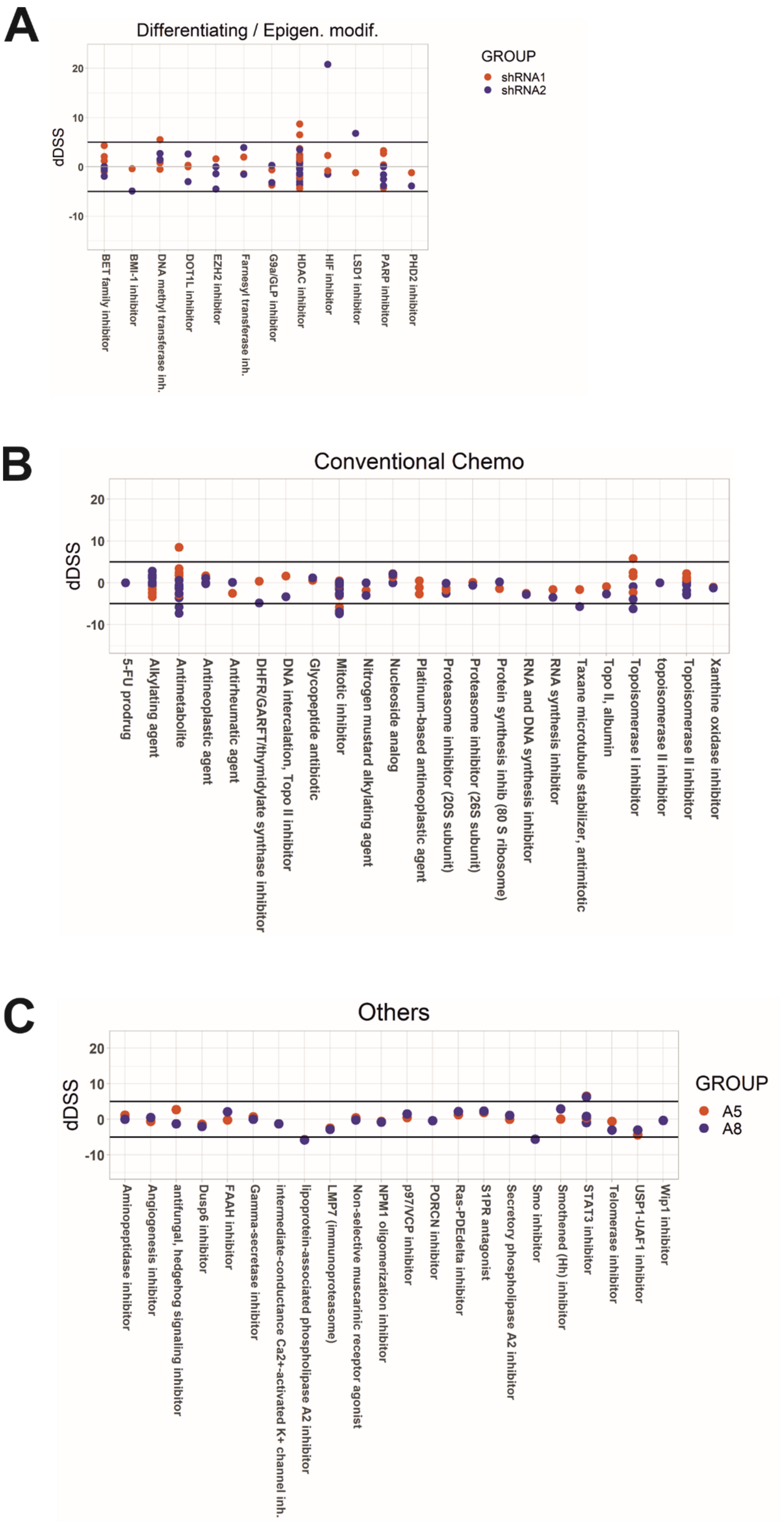

A
Differentiating / Epigen. modif.
dDSS
20
10
0
-10
GROUP
shRNA1
shRNA2
BET family inhibitor
BMI-1 inhibitor
DNA methyl transferase inh.
DOT1L inhibitor
EZH2 inhibitor
Farnesyl transferase inh.
G9a/GLP inhibitor
HDAC inhibitor
HIF inhibitor
LSD1 inhibitor
PARP inhibitor
PHD2 inhibitor
B
Conventional Chemo
dDSS
20
10
0
-10
5-FU prodrug
Alkylating agent
Antimetabolite
Antineoplastic agent
Antirheumatic agent
DHFR/GARFT/thymidylate synthase inhibitor
DNA intercalation, Topo II inhibitor
Glycopeptide antibiotic
Mitotic inhibitor
Nitrogen mustard alkylating agent
Nucleoside analog
Platinum-based antineoplastic agent
Proteasome inhibitor (20S subunit)
Proteasome inhibitor (26S subunit)
Protein synthesis inhib (80 S ribosome)
RNA and DNA synthesis inhibitor
RNA synthesis inhibitor
Taxane microtubule stabilizer, antimitotic
Topo II, albumin
Topoisomerase I inhibitor
topoisomerase II inhibitor
Topoisomerase II inhibitor
Xanthine oxidase inhibitor
C
Others
dDSS
20
10
0
-10
GROUP
A5
A8
Aminopeptidase inhibitor
Angiogenesis inhibitor
antifungal, hedgehog signaling inhibitor
Dusp6 inhibitor
FAAH inhibitor
Gamma-secretase inhibitor
intermediate-conductance Ca2+-activated K+ channel inh.
lipoprotein-associated phospholipase A2 inhibitor
LMP7 (immunoproteasome)
Non-selective muscarinic receptor agonist
NPM1 oligomerization inhibitor
p97/VCP inhibitor
PORCN inhibitor
Ras-PDEdelta inhibitor
S1PR antagonist
Secretory phospholipase A2 inhibitor
Smo inhibitor
Smothened (Hh) inhibitor
STAT3 inhibitor
Telomerase inhibitor
USP1-UAF1 inhibitor
Wip1 inhibitor

Figure S2. Effect of SND1 silencing (shRNA1 and shRNA2) to differentiating/epigenetic modification (a) or conventional chemotherapeutics related signaling pathways (b), and other cancer related signaling pathways (c). dDSS values above 5 are considered as a significant change.

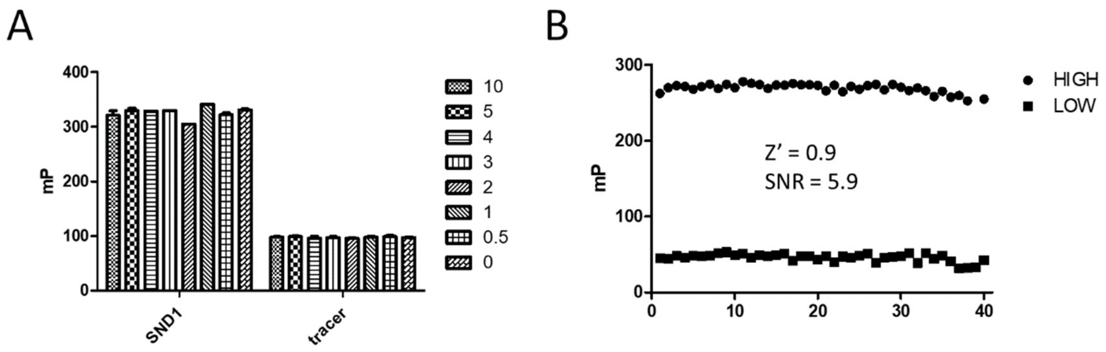

Figure S3. (a) DMSO tolerance of the FP screening assay using 0-10 % DMSO. SND1: 200 nM SND1 and 3 nM tracer, tracer: 3 nM tracer. **(b)** Assay validation using 40 maximal and minimal signal points. Signal-to-noise ratio (SNR) and screening window coefficient (Z′) are shown.

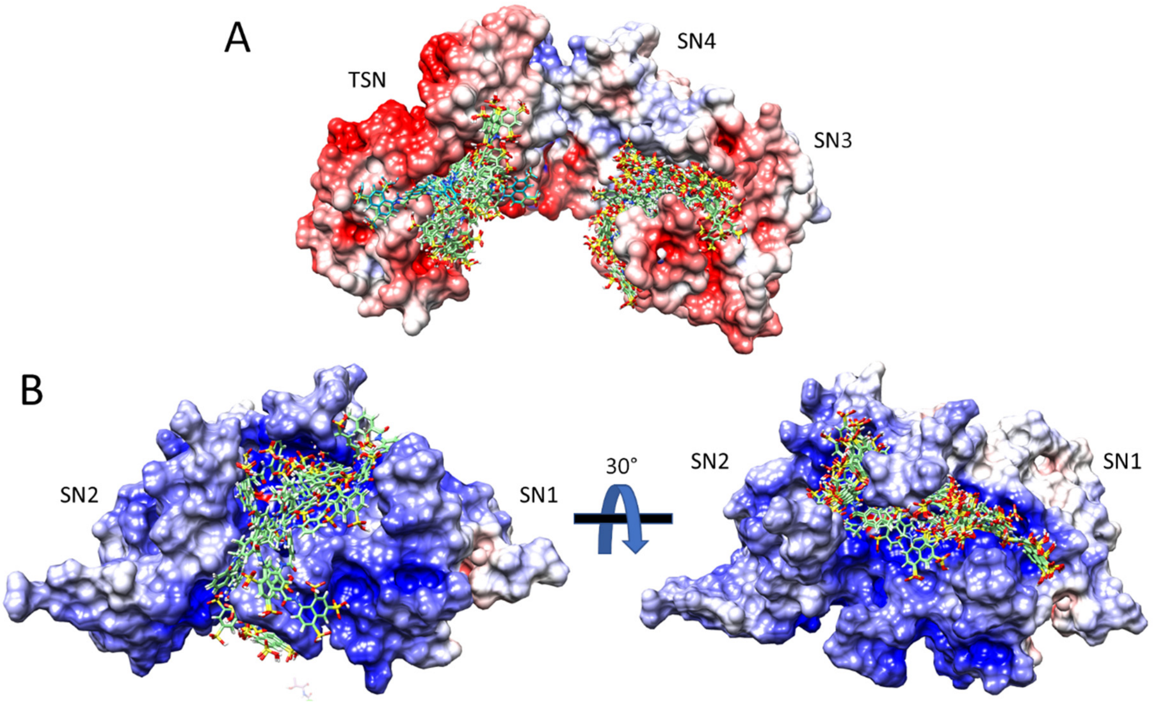

Figure S4. Molecular docking of suramin. **(a)** Suramin docked to SN3-SN4-TSN (pdb code 3bdl). Two clusters of docked poses are present, one in SN3 and the other in TSN domain. **(b)** Suramin docked to SN1-SN2 (pdb code 4wmg). On the left: cluster of poses at SN1-SN2 interface. On the right: cluster of poses perpedicular to SN1-SN2 interface.

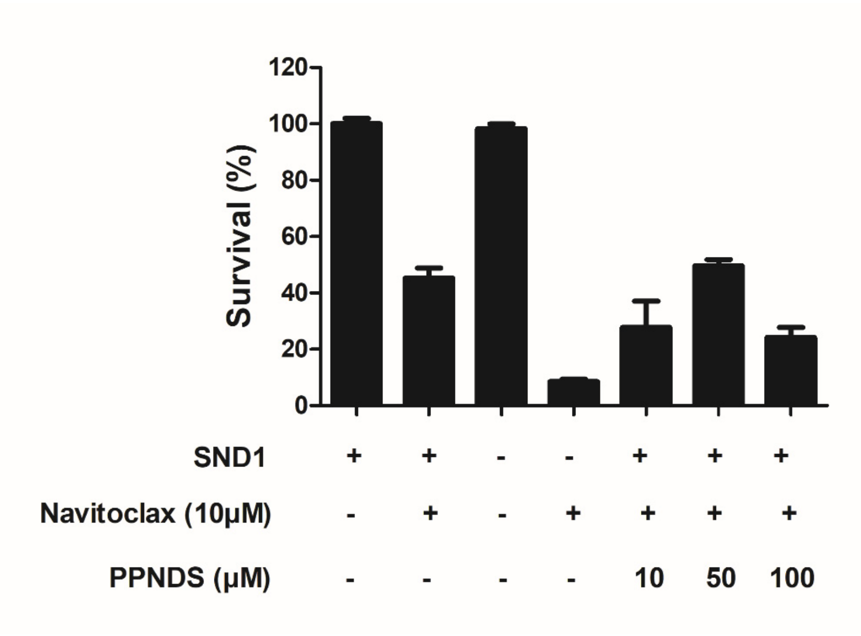

Figure S5. PPDNS does not cause synergetic reduction to cell viability together with navitoclax treatment.

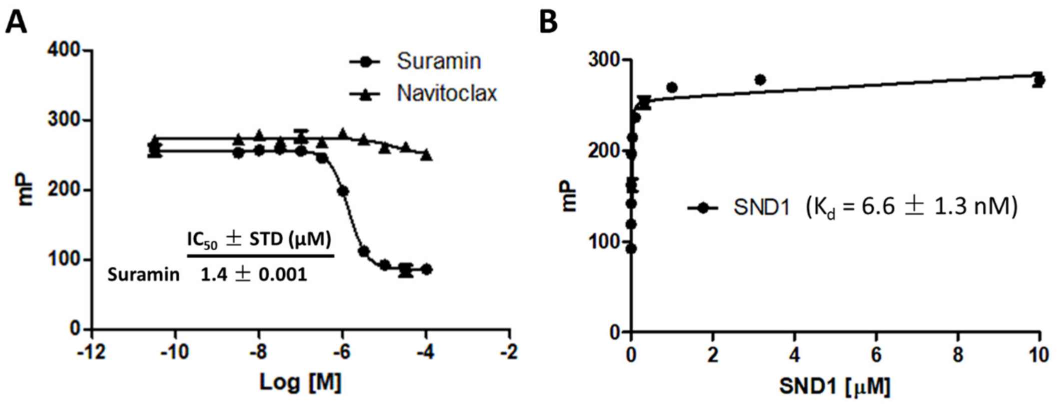

Figure S6. Fluorescence polarization assay using the miR-1-3p FP tracer. (a) Potencies of suramin and navitoclax against SND1-miR-1-3p interaction. (b) Binding affinity of SND1 to miR-1-3p.

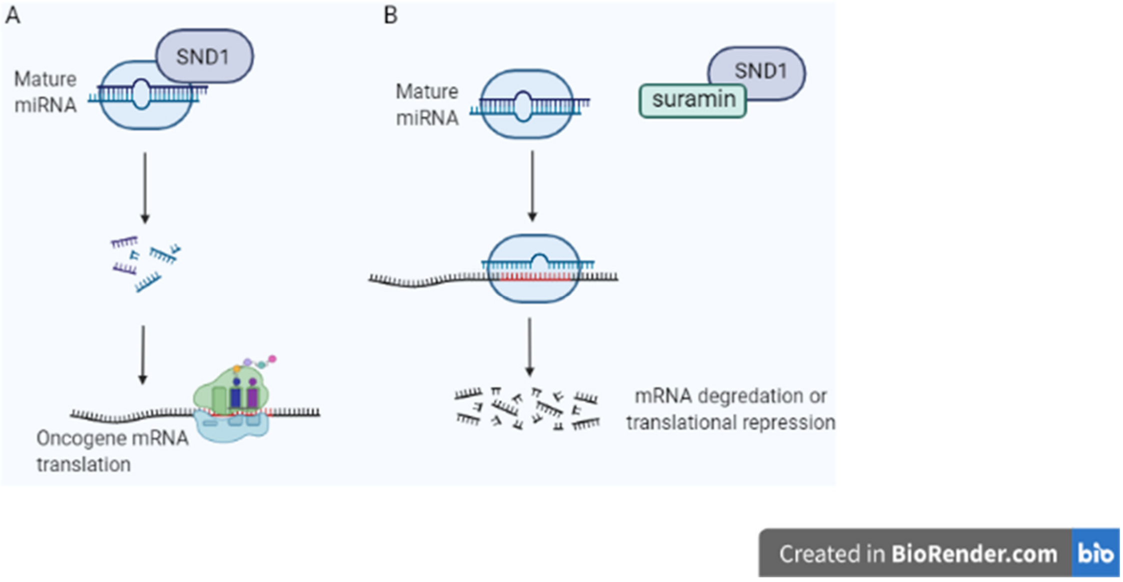

Figure S7. Schematic presentation of the function of SND1-miRNA interaction and the effect of suramin. **(**A) In cancer cells, SND1 (often overexpressed) binds to tumor suppressor miRNAs (e.g. miR-1-3p), and cleavages the miRNA from CA and/or UA sites. The translation of the target of the miRNA is not suppressed. (B) If suramin is present, it binds to RNA binding site of SND1 and prevents the miRNA binding and degradation. Tumor suppressor miRNA (miR-1-3p) is able to bind into its target and prevent its activity.
